# Dr. Lewis Wheeler Rose

**Published:** 1911-10

**Authors:** 


### OBITUARY

Dr. Lewis Wheeler Rose, of Rochester, died August 29, 1911, of heart disease, aged 45. He was a graduate of New York University, 1887, and a member of the A.M.A. and affiliated organizations, of the N. Y. State Pathologic Society, etc. He had been surgeon to the Soldiers' and Sailors' Home at Bath and, from 1892 to 1898 physician to the State Industrial Home. He was on the Surgical Staff of the Rochester City Hospital and had been President of the Medical Society of the County of Monroe.

